# Supplementary material for: Foliar Traits Drive Chlorophyll Fluorescence Variability in Chilean Sclerophyllous Species Under Early Outplanting Stress
Source: Plants (Basel). 2025 Aug 27;14(17):2682. doi: 10.3390/plants14172682 (PMC12430304; doi:10.3390/plants14172682)

Supplementary Information

Figure S1. Visual representations of morphological characteristics of leaves in the species under study. Compound leaves of *V. caven* (A), leaf rolling in *E. pulverulenta* (B) and *P. boldus* (C), V-shaped leaves in *L. caustica* (D), leaves of *Q. saponaria* (E), and *C. odorifera* (F). Photos were taken in one- and two-year-old seedlings of the experiment.

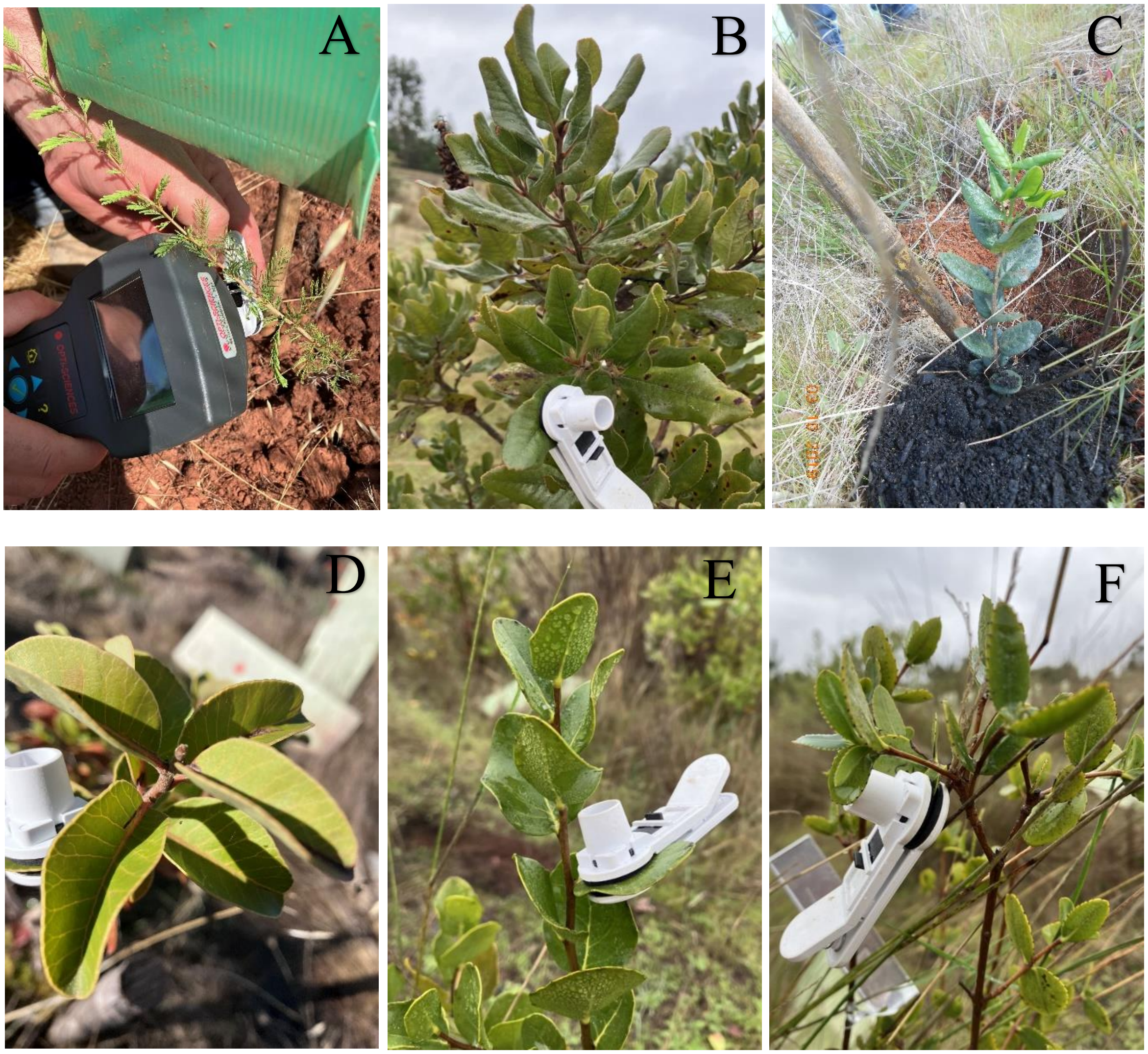

Supplement: Supplementary file 1 [file plants-14-02682-s001.zip › plants-3802023-supplementary.pdf]
